# Assessment of HIV knowledge and its determinants among the general population in Saudi Arabia: a national cross-sectional study

**DOI:** 10.1186/s12889-025-25401-5

**Published:** 2025-11-21

**Authors:** Najim Z. Alshahrani, Sultan M. Alshahrani

**Affiliations:** 1 Department of Family and Community Medicine, College of Medicine, University of Jeddah, Jeddah, Saudi Arabia; 2 College of Pharmacy, King Khalid University, Abha, Saudi Arabia

**Keywords:** HIV knowledge, Saudi arabia, Public health, HIV awareness, Stigma, Prevention strategies

## Abstract

**Background:**

Human Immunodeficiency Virus (HIV) remains a global health challenge, with Saudi Arabia reporting one of the lowest prevalence rates worldwide. However, societal stigma and cultural sensitivities may impact reporting and prevention efforts. Understanding HIV knowledge among the general population is crucial for developing effective public health strategies. This study aimed to assess the level of HIV knowledge among the general population in Saudi Arabia and examine the factors influencing it.

**Methods:**

A cross-sectional study was conducted using an online survey distributed through social media platforms. The validated Arabic version of the Brief HIV Knowledge Questionnaire (HIV-KQ-18) was used to assess HIV knowledge among 268 adult residents in Saudi Arabia. Participants were categorized into “Good knowledge” and “Poor knowledge” groups based on median score. Multivariate logistic regression was performed to identify predictors of good HIV knowledge.

**Results:**

Overall, 44.4% of participants demonstrated good HIV knowledge. Male participants showed significantly higher good HIV knowledge compared to females (61% vs. 39%, *p* < 0.001). Medical field participants exhibited significantly higher median knowledge scores than non-medical participants (9 vs. 6, *p* < 0.001). Multivariate analysis identified male gender (AOR = 2.27, 95% CI: 1.32–3.95, *p* = 0.003) and field of work/study as significant predictors, where non-medical participants were less likely to have good HIV knowledge compared to medical participants (AOR = 0.255, 95% CI: 0.13–0.47, *p* < 0.001). Educational level and employment status showed trends toward significance but were not independent predictors.

**Conclusions:**

Significant disparities in HIV knowledge exist across demographic and professional groups in Saudi Arabia, with gender and medical background emerging as key predictors. These findings highlight the need for targeted educational interventions, particularly for women and non-medical professionals. Implementation of comprehensive public health campaigns and integration of HIV education into various educational and professional settings are recommended to improve overall HIV awareness and prevention efforts.

## Background

The global health landscape has long been shaped by the challenges posed by Human Immunodeficiency Virus (HIV) and Acquired Immune Deficiency Syndrome (AIDS). By 2023, approximately 39.9 million individuals were living with HIV worldwide, with an estimated 630,000 deaths attributed to AIDS-related illnesses that year [1]. An estimated 0.8% of adults aged 15 to 49 years worldwide are living with HIV, which is considerably higher than the average prevalence in the Middle East and North Africa (MENA) region, where adult prevalence remains below 0.1% in most countries [2].

In Saudi Arabia, the first documented case of HIV was reported in 1984 [3], followed by a steady increase in cases until 2006, when the trend stabilized. As of the end of 2018, 6,744 individuals in the country were living with diagnosed HIV infection [4]. Although Saudi Arabia reports one of the lowest HIV prevalence rates globally [5], it is believed that the actual figures could be higher due to delays in reporting, impacted by societal stigma, cultural sensitivities, and religious factors [6]. Efforts to combat the spread of HIV are coordinated internationally through partnerships between the Joint United Nations Program on HIV/AIDS (UNAIDS), governments, and non-governmental organizations [7]. These collaborations aim to minimize infection rates and provide essential medications to those already affected.

The Gulf Cooperation Council (GCC), which includes Bahrain, Kuwait, Oman, Qatar, Saudi Arabia, and the United Arab Emirates, shares common socio-cultural and religious characteristics, with Islam as the dominant religion and Arabic as the primary language [8]. This region’s conservative social norms significantly influence discussions about sexual health and HIV. In 2021, over 42,000 people living with HIV resided in the GCC, with higher prevalence rates observed among specific at-risk groups such as injection drug users, female sex workers, and men who have sex with men [9].

Although non-Saudi residents historically accounted for most HIV cases, surveillance data from 2000 to 2009 show the contribution of Saudi nationals to total HIV notifications increased from approximately 20% in the early 2000 s to nearly 40% by 2009 [6]. Advances in antiretroviral therapy have drastically reduced HIV-related mortality [10, 11], and improved the quality of life for affected individuals. Highly active antiretroviral therapy (HAART) not only diminishes the risk of perinatal transmission but also lowers the general transmission risk [12, 13]. Consequently, in high-resource settings with consistent access to antiretroviral therapy, HIV/AIDS is now often regarded as a manageable chronic condition rather than a fatal disease, reflecting a paradigm shift in its management over recent decades.

Several studies have assessed HIV-related knowledge and attitudes within Saudi Arabia, revealing significant knowledge gaps and prevalent stigma. A recent nationwide cross-sectional study among 1,213 medical students found that while clinical-year students had better HIV knowledge, many respondents held misconceptions about mother-to-child and casual contact transmission [14]. These misconceptions were significantly associated with negative attitudes toward people living with HIV. Similarly, a systematic review of 19 studies on Saudi healthcare professionals and students reported widespread misinformation, unsatisfactory knowledge levels, and stigmatizing behaviors [15]. Among the general population, a large-scale study involving over 2,000 participants found high knowledge of transmission modes (mean score 84.2%) but low awareness and poor attitudes toward people with HIV (mean score 50%) [16]. These findings highlight the urgent need for evidence-based educational programs and stigma-reduction initiatives across both clinical and community settings.

Despite the availability of treatment and global efforts to control HIV, primary prevention remains a cornerstone in reducing new infections, particularly in settings where stigma and misinformation persist. In Saudi Arabia, evidence indicates that both healthcare professionals and the general public continue to harbor misconceptions about HIV transmission, often accompanied by negative attitudes toward people living with HIV. Understanding the public’s level of knowledge is therefore essential for guiding effective, culturally appropriate awareness and prevention strategies. This study was designed to assess HIV-related knowledge among the general population in Saudi Arabia, with the aim of generating evidence to support targeted public health interventions and education campaigns.

## Methods

### Study population

The study was conducted among the general adult population residing in Saudi Arabia in 2024. Eligible participants were Saudi and non-Saudi residents aged 18 years and above, with access to the internet and able to read and respond voluntarily to an online questionnaire.

### Sample size calculation

The sample size was determined using the sample size formula for estimating a proportion in a finite population. The total adult population of Saudi Arabia is estimated to exceed 26 million as of 2024. For the purpose of this study, a conservative estimate was used to ensure generalizability. While a previous study [17] reported an HIV knowledge proportion (P) of 77.4%, a more conservative value of *P* = 0.50 was applied in the final sample size calculation to maximize the required sample and account for variability. Using a 95% confidence level (Z = 1.96), a 5% margin of error (d = 0.05), and assuming a design effect (DEFF) of 1, the sample size was calculated using OpenEpi version 3.01 (https://www.openepi.com/SampleSize/SSPropor.htm) [18]. The final required sample size was 384 participants.

A total of 384 individuals initiated the survey through the online platform. The final analytical sample consisted of 268 participants who fully completed the questionnaire and provided explicit informed consent for their data to be utilized. This process resulted in an exclusion rate of 30.2%. It is critical to note that this figure primarily reflects participants who dropped off after viewing the introduction or consent form and did not grant permission to use their responses. This high non-consent rate is commonly observed in online research concerning sensitive health topics like HIV, and is likely exacerbated by the social and cultural stigma associated with HIV/AIDS research in Arab countries.

To ensure data quality and integrity, the Google Forms survey was configured to require responses for all items in the HIV Knowledge Questionnaire (HIV-KQ-18) before submission. Consequently, the final dataset included only fully completed questionnaires from consenting individuals. This design choice minimized item non-response and eliminated the need for data imputation in the subsequent statistical analysis.

## Sampling method

A non-probability convenience sampling technique was used. The survey was distributed via social media platforms (Twitter/X, Snapchat, WhatsApp, Facebook) and through email networks. Each invitation included a brief description of the study objectives, eligibility criteria, and informed consent. Participation was voluntary and anonymous.

### Study tools

The study utilized the Brief HIV Knowledge Questionnaire (HIV-KQ-18), developed by Carey and Schroder (2002) [19]. Due to the high sensitivity of the topic within the Saudi culture, the study included few sociodemographic data such as age, sex, and educational level.

The validated instrument consists of 18 items measuring knowledge about HIV transmission, prevention, and misconceptions. Each item has three possible responses: “True,” “False,” or “Don’t Know.” Correct answers are scored as 1, while incorrect and “Don’t Know” responses are scored as 0, resulting in a total possible score range of 0–18. The Arabic version validated by Terra et al. (2023) was used in this survey [20]. The Arabic version has demonstrated good internal consistency (KR-20 = 0.73). In terms of how well experts agreed on the clarity and relevance of each item, the item-level content validity index (I-CVI) ranged from 0.91 to 1.0, showing strong agreement. Similarly, the expert-level index (E-CVI), which reflects agreement among multiple reviewers, ranged from 0.92 to 1.0. Overall, the entire scale demonstrated excellent content validity, with a scale-level index (S-CVI) of 0.98 for both clarity and relevance.

### Data collection approach

Data was collected through Google Forms. The survey comprised three sections: Informed consent, demographic information (age, gender, education level, occupation, etc.), and the Arabic version of HIV-KQ-18 questionnaire. The survey was available in Arabic. Data collection took place for 5 months (July- November) in 2024. To ensure data integrity, measures were implemented to prevent multiple submissions from the same participant by restricting responses to one submission per email address linked to the Google Form account.

### Statistical analysis

Data analysis was performed using RStudio. Data cleaning and preparation were conducted using the tidyverse package. Descriptive statistics were computed: categorical variables were summarized as frequencies and percentages, and continuous variables were presented as medians and interquartile ranges (IQRs). The total HIV knowledge score (0–18) was computed for each participant. To categorize knowledge levels, the median score of the sample was used as a cutoff (Median Split): participants scoring above the median were classified as having “Good knowledge”, and those scoring at or below were classified as having “Poor knowledge”. This approach has been commonly applied in previous knowledge and awareness studies as a practical way to distinguish higher versus lower knowledge groups [21]. It was chosen due to the non-normal distribution of the score and the absence of a universally validated cut-off for HIV knowledge in the Saudi or Middle Eastern context. While this method may reduce variability and statistical power, it provides clear interpretability and facilitates straightforward comparisons of determinants across groups [21]. Normality was assessed using the Shapiro-Wilk test and histogram visualizations. As the data were not normally distributed, non-parametric tests were applied. Chi-square tests were used to assess associations between categorical variables and knowledge levels, while Mann-Whitney U tests were used to compare continuous variables. Multivariable logistic regression analysis was conducted to identify predictors of good HIV knowledge. Adjusted odds ratios (AORs) and 95% confidence intervals (CIs) were reported. Data visualization was performed using the ggplot2 and ggpubr packages, and results were summarized using gtsummary and broom. A two-tailed p-value of < 0.05 was considered statistically significant.

## Results

### Participant characteristics and HIV knowledge

A total of 268 participants completed the survey, with 55.6% (*n* = 149) demonstrating poor HIV knowledge and 44.4% (*n* = 119) demonstrating good HIV knowledge (Table 1). The median age of participants was 24 years (IQR: 22–27) for those with poor knowledge and 23 years (IQR: 21–31) for those with good knowledge, with no significant difference between the two groups (*p* = 0.823). Significant differences were observed in the distribution of HIV knowledge based on sex. Among participants with good HIV knowledge, 61% were males, while only 39% were females (*p* < 0.001). In contrast, among those with poor HIV knowledge, 59% were females and 41% were males. Marital status also showed differences, with 64% of participants with good knowledge being single, compared to 52% of those with poor knowledge (*p* = 0.037). Educational level was another determinant of HIV knowledge. Among participants with good knowledge, 19% had postgraduate degrees, compared to 6.7% of those with poor knowledge (*p* = 0.005). Employment status also played a role, with 38% of participants with good knowledge being employed, compared to 29% of those with poor knowledge (*p* < 0.001). Additionally, 61% of participants with good knowledge were in the medical fields, while only 26% of those with poor knowledge were in the medical fields (*p* < 0.001). No significant differences were observed for nationality, chronic disease status, or smoking status (*p* > 0.05).

**Table 1:** Participants’ characteristics regarding HIV knowledge

Variable		Poor HIV Knowledge *n* (%)	Good HIV Knowledge *n* (%)	*P*-value^1^
Overall (N)		149 (55.6%)	119 (44.4%)	
Age	Median (Q1-Q3)	24 (22, 27)	23 (21, 31)	0.823
Sex	Female	88 (65.2%)	47 (34.8%)	< 0.001*
	Male	61 (45.9%)	72 (54.1%)	
Marital status	Single	78 (50.6%)	76 (49.4%)	0.037*
	Engaged	29 (74.4%)	10 (25.6%)	
	Married	38 (58.5%)	27 (41.5%)	
	Divorced	4 (40.0%)	6 (60.0%)	
Nationality	Saudi	143 (55.4%)	115 (44.6%)	0.775
	Non-Saudi	6 (60.0%)	4 (40.0%)	
Education level	Pre-university	29 (65.9%)	15 (34.1%)	0.005*
	University	110 (57.6%)	81 (42.4%)	
	Postgraduate	10 (30.3%)	23 (69.7%)	
Employment status	Employed	43 (48.9%)	45 (51.1%)	< 0.001*
	Unemployed	44 (77.2%)	13 (22.8%)	
	Student	62 (50.4%)	61 (49.6%)	
Field of study	Medical	39 (34.8%)	73 (65.2%)	< 0.001*
	Non-Medical	110 (70.5%)	46 (29.5%)	
Chronic disease	Yes	45 (51.1%)	43 (48.9%)	0.304
	No	104 (71.7%)	76 (28.3%)	
Current smoking	Yes	24 (51.1%)	23 (48.9%)	0.490
	No	125 (64.2%)	96 (35.8%)	

1 Wilcoxon rank sum test; Pearson’s Chi-squared test; Fisher’s exact test

*Statistically significant P-value < 0.05

### HIV knowledge assessment by field of work/study

HIV knowledge assessment by field of work/study highlighted distinct differences between medical and non-medical participants (Table 2). Medical participants consistently outperformed their non-medical counterparts in understanding HIV transmission and prevention. For instance, they were more likely to correctly identify that coughing and sneezing do not spread HIV (76% vs. 36%, *p* < 0.001) and that showering after sex does not prevent HIV infection (59% vs. 37%, *p* < 0.001). Additionally, medical participants demonstrated greater awareness regarding the ineffectiveness of natural skin condoms compared to latex condoms (21% vs. 15%, *p* = 0.214), although this difference was not statistically significant. Some questions, such as the transmission of HIV through pregnancy (19% vs. 21%, *p* = 0.721) and the existence of a female condom (24% vs. 23%, *p* = 0.844), did not reveal significant differences between the two groups.

**Table 2:** HIV knowledge assessment by medical and non-medical participants

*N*.	Question	Correct *n* (%)^1^		*P*-value^2^
		Medical *N* = 112	Non-Medical *N* = 156	
1	Coughing and sneezing DO NOT spread HIV.	85 (76%)	56 (36%)	< 0.001*
2	A person can get HIV by sharing a glass of water with someone who has HIV.	81 (72%)	62 (40%)	< 0.001*
3	Pulling out the penis before a man climaxes/cums keeps a woman from getting HIV during sex.	55 (49%)	45 (29%)	< 0.001*
4	A woman can get HIV if she has anal sex with a man	71 (63%	63 (40%)	< 0.001*
5	Showering, or washing one’s genitals/private parts, after sex keeps a person from getting HIV.	66 (59%)	58 (37%)	< 0.001*
6	All pregnant women infected with HIV will have babies born with AIDS.	21 (19%)	32 (21%)	0.721
7	People who have been infected with HIV quickly show serious signs of being infected.	61 (54%)	48 (31%)	< 0.001*
8	There is a vaccine that can stop adults from getting HIV.	55 (49%)	44 (28%)	< 0.001*
9	People are likely to get HIV by deep kissing, putting their tongue in their partner’s mouth, if their partner has HIV.	53 (47%)	49 (31%)	0.008*
10	A woman cannot get HIV if she has sex during her period.	61 (54%)	60 (38%)	0.009*
11	There is a female condom that can help decrease a woman’s chance of getting HIV.	27 (24%)	36 (23%)	0.844
12	A natural skin condom is more protective against HIV than a latex condom.	23 (21%)	23 (15%)	0.214
13	A person will NOT get HIV if she or he is taking antibiotics.	61 (54%)	44 (28%)	< 0.001*
14	Having sex with more than one partner can increase a person’s chance of being infected with HIV.	98 (88%)	118 (76%)	0.015*
15	Taking a test for HIV one week after having sex will tell a person if she or he has HIV.	37 (33%)	30 (19%)	0.010*
16	A person can get HIV by sitting in a hot tub or a swimming pool with a person who has HIV.	51 (46%)	57 (37%)	0.138
17	A person can get HIV from oral sex.	52 (46%)	54 (35%)	0.051
18	Using Vaseline or baby oil with condoms lowers the chance of getting HIV.	44 (39%)	48 (31%)	0.147

^1^ Median (Q1, Q3); n (%)

^2^ Wilcoxon rank sum test; Pearson’s Chi-squared test; Fisher’s exact test

*Statistically significant P-value < 0.05

### Distribution of total HIV knowledge scores by field of work/study

**Fig. 1:**
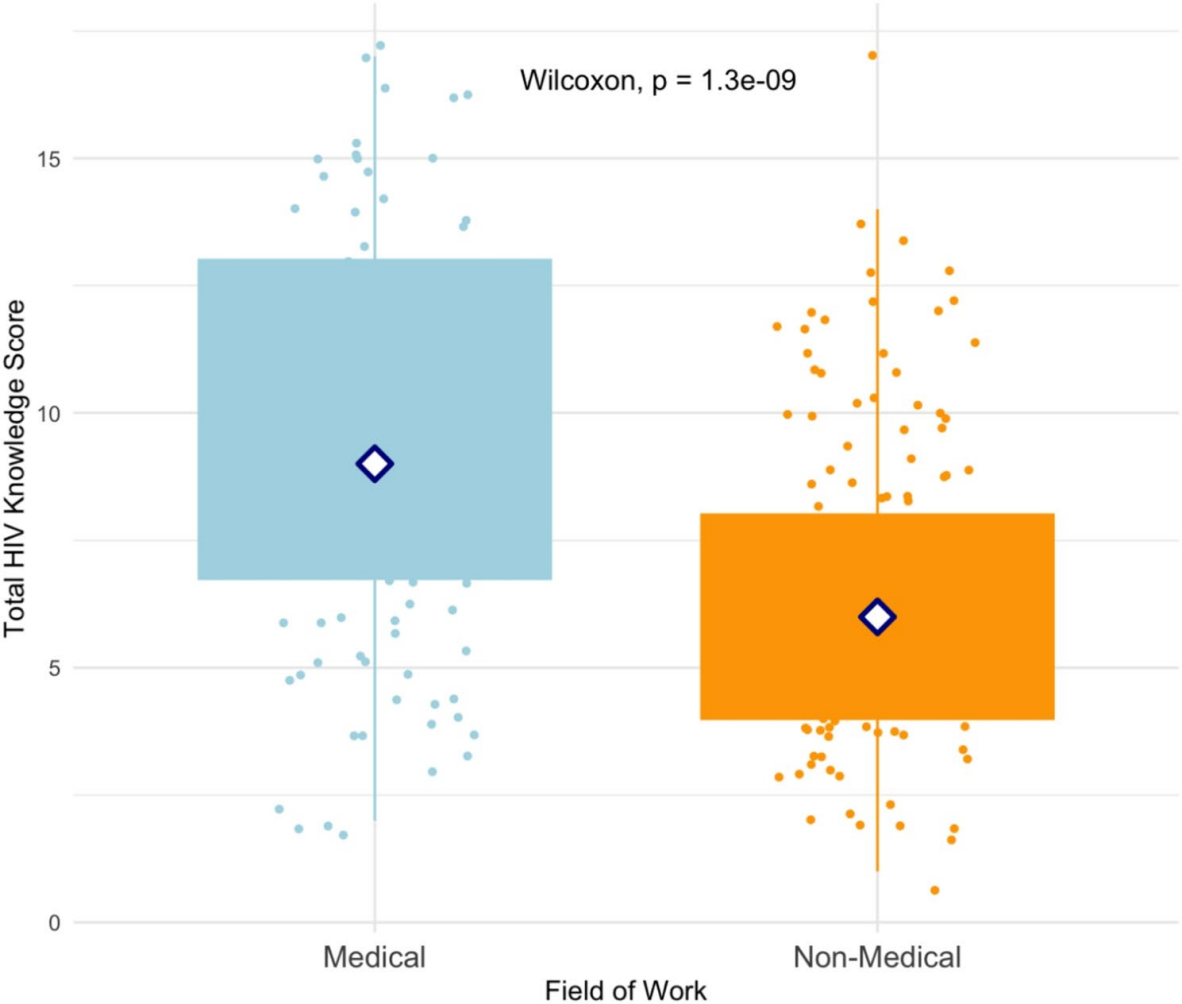
Box Plot of HIV knowledge total scores comparing medical and non-medical participants

Figure 1 presents the distribution of total HIV knowledge scores between medical and non-medical participants. Medical participants had significantly higher median scores (9, IQR: 6.5–13) compared to non-medical participants (6, IQR: 4–8, *p* < 0.001).

### Multivariate logistic regression of factors associated with good HIV knowledge

A multivariate logistic regression analysis was conducted to explore factors associated with good HIV knowledge (Table 3.). Male participants had significantly higher odds of having good HIV knowledge compared to females (Adjusted Odds Ratio [AOR] = 2.27, 95% CI: 1.32–3.95, *p* = 0.003). Additionally, individuals from non-medical fields were significantly less likely to report good HIV knowledge compared to those in medical fields (AOR = 0.25, 95% CI: 0.13–0.47, *p* < 0.001). Other variables, including marital status, educational level, and employment status, did not show statistically significant associations in the multivariate model, although some trends were observed.

**Table 3.** Multivariate logistic regression of factors influencing good HIV knowledge

Variables		AOR	P-value	Confidence Interval 95%	
				Lower	Upper
Sex	Female	Reference			
	Male	2.27^*^	0.003*	1.32	3.95
Marital status	Divorced	Reference			
	Engaged	0.26	0.095	0.05	1.23
	Married	0.36	0.188	0.07	1.59
	Single	0.59	0.489	0.12	2.55
Educational level	Postgraduate (Master’s or PhD)	Reference			
	Pre-university (Secondary or Other)	0.44	0.177	0.13	1.44
	University (Bachelor’s or Diploma)	0.46	0.124	0.17	1.23
Employment status	Employed	Reference			
	Student	0.65	0.287	0.29	1.43
	Unemployed	0.51	0.125	0.21	1.19
Field of work/study	Medical	Reference			
	Non-Medical	0.25	< 0.001*	0.13	0.47

*Statistically significant P-value < 0.05

## Discussion

The findings of this study provide valuable insights into the current state of HIV knowledge among the general population in Saudi Arabia, revealing significant disparities across various demographic and professional groups and underscoring the need for targeted interventions to improve public awareness.

In our study, male participants had significantly higher odds of possessing HIV knowledge compared to their female counterparts, a trend that aligns with previous studies highlighting gender disparities in health-related awareness [22]. This discrepancy may be influenced by cultural norms and unequal access to health information, underscoring the need for targeted interventions to address these gaps [23]. While bivariate analysis suggested that single individuals had better HIV knowledge than married or other groups, this association was not statistically significant in the multivariate analysis. Therefore, this trend should be interpreted cautiously and may reflect differences in exposure to health messaging or life stage priorities rather than a robust association.

Although educational attainment and employment status appeared to be associated with HIV knowledge in bivariate analyses, these variables were not statistically significant in the adjusted model. Participants with postgraduate degrees showed a higher proportion of good knowledge, followed by those with university and pre-university education. These results are consistent with global trends indicating a strong correlation between higher education and improved health literacy [24]. A previous study in China showed that there were statistically significant differences between subgroups classified by age, education, and occupation [25]. This suggests that integrating comprehensive HIV education into school curricula and continuing education programs could play a pivotal role in bridging existing knowledge gaps [26].

Employment status was also associated with HIV knowledge at the bivariate level, with employed individuals and students demonstrating better knowledge compared to unemployed participants. Employment often provides access to workplace health programs, which may explain this disparity and highlight the potential benefits of incorporating HIV education into workplace wellness initiatives [27]. Participants from medical fields demonstrated notably higher HIV knowledge than those from non-medical backgrounds, an expected finding given the detailed education on infectious diseases provided during medical training. However, even among medical professionals, gaps in specific areas, such as the effectiveness of natural skin condoms, were identified, emphasizing the importance of continuous education and professional development [28].

Multivariate logistic regression analysis further revealed that being male and working or studying in a medical field were significantly associated with good HIV knowledge. Male participants had more than twice the odds of reporting good knowledge, while non-medical participants were significantly less likely to have good knowledge compared to their medical counterparts. These findings underscore the necessity of tailoring educational efforts based on demographic and professional characteristics to maximize impact [29, 30].

The study identified several persistent misconceptions and gaps in HIV knowledge, particularly regarding modes of transmission and prevention. A significant proportion of participants held incorrect beliefs, such as the idea that HIV can be transmitted through casual contact, including sharing glasses of water (36% of non-medical participants answered correctly) or sitting in hot tubs or swimming pools with someone who has HIV (37% of non-medical participants answered correctly). Additionally, many participants mistakenly believed that antibiotics could prevent HIV (28% of non-medical participants answered correctly) or that pulling out the penis before climax prevents transmission (29% of non-medical participants answered correctly). These misconceptions were significantly more prevalent among non-medical participants compared to those in medical fields, highlighting a critical gap in public understanding of HIV. Such misunderstandings can contribute to unnecessary fear, stigma, and discriminatory behaviors toward people living with HIV, as well as risky behaviors that increase the likelihood of transmission [31, 32].

The results of this study are consistent with earlier research conducted both locally and globally, which has consistently highlighted insufficient public awareness about HIV/AIDS and its transmission routes [25, 33, 34]. For instance, a study conducted in northern Saudi Arabia documented significant gaps in public understanding of HIV/AIDS, particularly among non-medical populations [22, 23]. Similarly, misconceptions about casual contact as a mode of transmission have been noted among healthcare workers and students in Saudi Arabia, reinforcing the need for targeted educational initiatives [35]. These findings collectively emphasize the urgency of implementing comprehensive public health campaigns aimed at improving HIV awareness across all demographic groups in Saudi Arabia.

### Implications for public health policy

In light of these findings, several implications for public health policy and practice can be drawn. Gender-specific interventions should be developed to address the lower levels of HIV knowledge observed among women, ensuring they have equitable access to accurate information. Educational programs should focus on pre-university populations and non-medical professionals to bridge existing gaps, potentially through the integration of HIV education into school curricula and vocational training programs. Workplace health initiatives should also be expanded to include HIV education, capitalizing on the higher knowledge levels observed among employed individuals. Additionally, medical curriculum enhancements are necessary to address gaps in specific areas of HIV knowledge among medical professionals, ensuring they remain up to date with the latest advancements in HIV management and prevention.

### Study limitations

Several limitations must be considered when interpreting the findings of this study. The sampling and generalizability limitation is the most critical and requires immediate emphasis. The reliance on convenience sampling through digital channels introduced a severe selection bias, resulting in a sample that is primarily younger, urban, and more educated. This means the findings cannot be generalized to the entire population, especially older adults, rural communities, or those with limited internet access. This non-representativeness must be clearly stated, requiring caution when interpreting the external validity of the results.

The high non-response and sample size issue further compromises the study’s rigor. The reported 30.2% non-response rate contributed to the final sample size falling short of the calculated target. This drop-off may have introduced an additional selection bias, possibly excluding individuals less comfortable discussing sensitive topics like HIV. Future methodology should focus on addressing participant fatigue and sensitivity to improve completion rates and reduce this source of bias.

Regarding the study design and causality, the cross-sectional nature of the study limits the ability to establish causal relationships or monitor how HIV knowledge changes over time. While suitable for a baseline assessment, the current design cannot determine if demographic factors directly cause the observed knowledge gaps or how effective future awareness campaigns might be. Therefore, the findings should be framed as a snapshot in time, and the need for subsequent longitudinal research must be clearly highlighted.

Finally, the measurement and response bias presents a distinct set of constraints. Despite using a validated instrument, the self-administered nature of the survey exposes the data to socially desirable responding or simple guessing, which may have led to an overestimation of actual knowledge levels. Additionally, the statistical decision to dichotomize the non-normally distributed knowledge scores—while aiding interpretation—may have oversimplified variability and potentially reduced the statistical power of the analysis. Future research should consider continuous analysis or the use of established, validated cut-off thresholds for greater precision.

## Conclusion

This cross-sectional study successfully provided a snapshot of HIV knowledge levels among a specific segment of the Saudi population. Based on the responses from our convenience sample largely recruited through online channels, the findings indicate significant disparities in HIV knowledge across demographic and professional groups, with notable gaps observed among those who are older, less educated, or employed in non-healthcare sectors.

However, the interpretation of these findings must be viewed within the context of several major limitations, including the non-representative convenience sampling and a high non-response rate. Our reliance on social media recruitment likely resulted in a sample skewed toward younger, urban, and more educated individuals, meaning the results may not generalize to the entire Saudi populace, particularly older adults or those in rural areas with limited internet access.

While causal inferences are limited by the study’s design, the observed knowledge gaps among our online-active cohort still highlight the urgent need for targeted educational interventions. Future public health efforts should focus on designing campaigns that can effectively reach and engage the underrepresented segments of the population that our methodology could not fully capture. Addressing these identified knowledge gaps, even within this specific demographic, is a crucial step toward reducing the persistent stigma associated with HIV/AIDS and promoting a more informed public health response in Saudi Arabia.

## Data Availability

The datasets used and/or analyzed during the current study are available from the corresponding author on reasonable request.
